# Maturation-Induced Cloaking of Neutralization Epitopes on HIV-1 Particles

**DOI:** 10.1371/journal.ppat.1002234

**Published:** 2011-09-08

**Authors:** Amanda S. Joyner, Jordan R. Willis, James E. Crowe, Christopher Aiken

**Affiliations:** 1 Department of Microbiology and Immunology, Vanderbilt University School of Medicine, Nashville, Tennessee, United States of America; 2 Chemical and Physical Biology Program, Vanderbilt University School of Medicine, Nashville, Tennessee, United States of America; 3 Department of Pediatrics, Vanderbilt University School of Medicine, Nashville, Tennessee, United States of America; University of Zurich, Switzerland

## Abstract

To become infectious, HIV-1 particles undergo a maturation process involving proteolytic cleavage of the Gag and Gag-Pol polyproteins. Immature particles contain a highly stable spherical Gag lattice and are impaired for fusion with target cells. The fusion impairment is relieved by truncation of the gp41 cytoplasmic tail (CT), indicating that an interaction between the immature viral core and gp41 within the particle represses HIV-1 fusion by an unknown mechanism. We hypothesized that the conformation of Env on the viral surface is regulated allosterically by interactions with the HIV-1 core during particle maturation. To test this, we quantified the binding of a panel of monoclonal antibodies to mature and immature HIV-1 particles by immunofluorescence imaging. Surprisingly, immature particles exhibited markedly enhanced binding of several gp41-specific antibodies, including two that recognize the membrane proximal external region (MPER) and neutralize diverse HIV-1 strains. Several of the differences in epitope exposure on mature and immature particles were abolished by truncation of the gp41 CT, thus linking the immature HIV-1 fusion defect with altered Env conformation. Our results suggest that perturbation of fusion-dependent Env conformational changes contributes to the impaired fusion of immature particles. Masking of neutralization-sensitive epitopes during particle maturation may contribute to HIV-1 immune evasion and has practical implications for vaccine strategies targeting the gp41 MPER.

## Author Summary

The conformation of HIV-1 Env is of tremendous importance from an immunological standpoint. While several human monoclonal antibodies that exhibit broadly neutralizing activity have been identified, efforts to elicit such antibodies have met with minimal success. Here, we show that the conformation of Env is altered on the surface of immature vs. mature HIV-1 particles in such a way that certain epitopes recognized by some broadly neutralizing antibodies are more exposed on immature virions. This maturation-dependent conformational masking may represent an important mechanism of HIV-1 immune evasion.

## Introduction

HIV-1 fusion is mediated by the Env glycoprotein, a trimeric complex of heterodimers composed of the surface glycoprotein (SU) gp120 and the transmembrane glycoprotein (TM) gp41. Fusion of virions with target cells takes place through a series of events initiated by binding of gp120 to CD4 on the surface of the target cell (reviewed in [1]). CD4 binding induces conformational changes in gp120 that permit exposure of the coreceptor-binding site, composed of the bridging sheet (consisting of four discontinuous anti-parallel beta strands) and the third hypervariable (V3) loop. Subsequent engagement of CD4-bound gp120 by a chemokine coreceptor—either CCR5 or CXCR4—triggers dramatic conformational changes in gp41 that result in fusion of viral and cellular membranes.

A common feature of lentiviruses is that their TM proteins have long cytoplasmic tails. HIV-1 gp41 encodes a 152 amino acid cytoplasmic tail (CT), while TM proteins of simple retroviruses have tails of 20–50 amino acids in length [2]. Several activities have been attributed to the gp41 CT, including polarized budding of HIV-1 particles from epithelial cell monolayers [3], rapid internalization of Env from the cell surface [4], [5], incorporation of Env into virions during particle assembly [6], [7], and interaction with Pr55^Gag^ during virion assembly [5], [6], [7], [8], [9].

To become infectious, newly formed HIV-1 particles must undergo a process of maturation involving specific cleavage of the major structural polyprotein Pr55^Gag^ by the viral protease. Immature HIV-1 particles contain stable cores and are non-infectious due to defects in early post-entry steps of the life cycle [10]. However, recent studies have demonstrated that immature virions are also impaired for fusion with target cells and that the gp41 CT plays a key role in repressing immature HIV-1 particle fusion [11], [12], [13].

The detailed mechanism by which HIV-1 fusion is regulated by structural changes within the core has not been determined, but one recent study attributed the repression to a change in physico-mechanical properties (*i.e.* “stiffness”) that accompanies HIV-1 maturation [14]. An alternative hypothesis is that maturation triggers a conformational change in the ectodomain of the Env glycoprotein complex, releasing it into a fusion-competent state. Such a mechanism might also limit the exposure of neutralization-sensitive epitopes in gp120 and gp41, thus promoting immune evasion. Previous work has revealed that the gp41 CT modulates Env conformation on HIV-1, HIV-2, and SIV, thus lending support to the latter hypothesis [15], [16], [17].

To test whether HIV-1 particle maturation alters the conformation of the Env proteins, we used a sensitive and quantitative imaging-based antibody-binding assay to probe the conformations of full-length and CT-truncated Env proteins on mature and immature HIV-1 particles. The results revealed specific epitopes in gp120 and gp41 that exhibit greater exposure on immature vs. mature virions, including two in the membrane-proximal external region (MPER). Therefore, Env trimers on immature virions are present in an exposed conformation, and neutralization-sensitive epitopes undergo conformational masking during particle maturation.

## Results

### Immunofluorescence-based detection of gp120 and gp41 epitopes on HIV-1 particles

**Figure 1:**
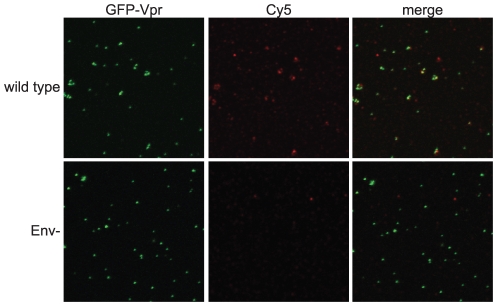
Quantitative imaging-based assay to detect Env conformations. HIV-1 virions containing a GFP-Vpr fusion protein were plated on glass cover slips, incubated with an Env-specific mAb, fixed, incubated with a Cy5-conjugated secondary antibody, and mounted. Samples were imaged using a Zeiss LSM 510 META inverted confocal microscope. Top row: wild-type HIV-1 particles; bottom row: Env negative HIV-1 particles.

To analyze Env conformation on HIV-1 particles, we developed a sensitive and quantitative imaging-based assay for binding of antibodies to virions. The assay was designed to permit the use of a panel of available conformation-specific monoclonal antibodies (mAbs) specific for gp120 and gp41. HIV-1 particles, containing a GFP-Vpr fusion protein, were immobilized on glass cover slips and stained using an indirect immunofluorescence protocol. To avoid potential artifacts resulting from fixation, Env-specific primary antibodies were bound under native conditions. After washing to remove unbound antibodies, antibody-bound virions were fixed with paraformaldehyde and detected by addition of a fluorescent, Cy5-conjugated secondary antibody. By this approach, the gp120-specific mAb 2G12 readily detected wild-type HIV-1 particles, as seen by the colocalization between the GFP-labeled virions and the Cy5 antibody fluorescence (Fig. 1, top row). As a control, Env-defective HIV-1 particles were not bound by 2G12, thus establishing the specificity of the assay (Fig. 1, bottom row).

The imaging-based assay detects antibody binding to individual HIV-1 particles, allowing for analysis of the distribution in staining intensities among a population of virions. The binding signal generally exhibited a broad distribution, with the bulk of the particles exhibiting low-level binding with a wide tail toward higher binding. Wild-type HIV-1 particles have been reported to contain approximately 10 Env trimers, with considerable variability among virions [18]. In the example shown for mAb 4E10 binding, a larger percentage of mature, wild type virions exhibited a lower average intensity than immature virions while more immature virions had high average intensities (Fig. S1A). The change in median average intensity per particle observed over the distribution was also apparent in the fluorescence images (Fig. S2). These are examples of data used to quantitatively compare the binding of antibodies to mature and immature HIV-1 particles. Representative histograms for each antibody are included in the supporting information.

As an additional control, the fluorescence distribution for Env-defective immature particles was determined for mAb 4E10 and mAb b12 binding. As expected, there was no difference between the binding to mature and immature particles lacking HIV-1 Env (Fig. S3), and the binding to each was minimal.

To facilitate interpretation of the particle imaging data, immunoblotting of viral lysates was performed to compare Env levels on virions (Fig. S4A). Quantification of band intensities revealed a difference of no more than 20% between mature and immature particles (Fig. S4B). Consistent with our previous work and that of others [8], [13], we observed higher TM/SU ratios on both mature and immature particles lacking the gp41 CT, owing to the elevated TM protein association with HIV-1 particles. Since our analyses focused mainly on pairwise comparisons between mature and immature particles, the elevated gp41 levels on CT-truncated particles does not represent a confounding factor. We conclude that the quantitative differences in immunodetection of Env on the surface of mature and immature HIV-1 particles result from differences in Env conformation.

### Env trimers are present in an altered conformation on immature HIV-1 particles

**Figure 2:**
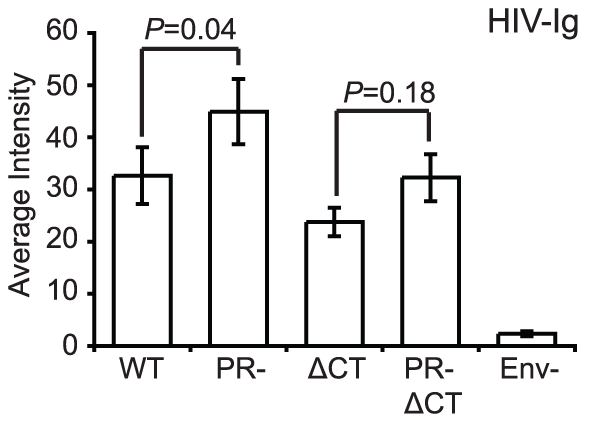
Binding of polyclonal human antibodies to HIV-1 virions. HIV-1 virions containing GFP-Vpr were treated as described in the legend to Figure 1. The intensity of Cy5 antibody staining to HIV-1 particles was analyzed with Metamorph. The data were compiled from four independent experiments where six independent fields containing on average 200–600 particles were evaluated for the median average intensity. N = 4; standard errors of the mean are shown. The data were analyzed using Wilcoxon rank-sum tests. WT: wild type HIV-1; PR: protease defective HIV-1; ΔCT: gp41 cytoplasmic tail-truncated HIV-1.

As an initial test to compare conformations of Env proteins on mature and immature virions, we tested the binding of HIV-Ig, a polyclonal IgG pool isolated from HIV-1 infected individuals. Under the optimized experimental conditions, Env-defective HIV-1 particles were not bound by HIV-Ig, indicating that staining was specific for Env (Fig. 2). We observed that the median staining intensity was 28% higher for immature versus mature particles. Immature HIV-1 particles lacking the gp41 cytoplasmic tail also exhibited elevated staining with HIV-Ig relative to the corresponding CT-truncated mature particles; however, this increase was not statistically significant. Because the imaging-based assay permits quantification of antibody binding to each particle, we also examined the distribution of HIV-Ig antibody binding (Fig. S5). Analysis of the distribution revealed that the bulk of the particles bound lower quantities of antibodies, with a tail toward higher binding levels. These results indicate that while some of the HIV-1 Env epitopes recognized by HIV-Ig exhibit enhanced exposure on immature particles, the gp41 CT is not required for the enhanced antibody binding. However, some antigenic elements on the surface of HIV-1 virions could be masked by cleavage of the Gag polyprotein inside the virions. Therefore, we asked whether specific Env epitopes differ between immature and mature virions and whether such differences depend on the gp41 CT.

### Conformation-independent antibodies directed toward gp120 are not altered during HIV-1 maturation

**Figure 3:**
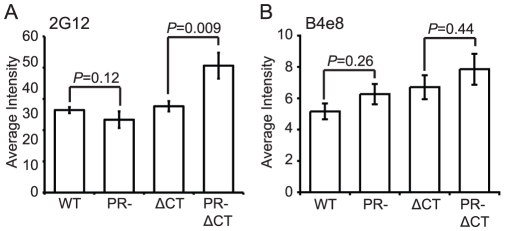
Analysis of exposure of the gp120 V3 loop and gp120 glycans on the surface of mature and immature HIV-1 particles. N = 3 (2G12 binding to ΔCT and PR-ΔCT), 4 (2G12 binding to WT and PR-, B4e8); error bars represent SEM. (A) mAb 2G12 binding; (B) mAb B4e8 binding.

MAb 2G12 specifically recognizes N-linked glycans in the C2, C3, C4, and V4 domains of gp120 [19]; binding of this antibody is thus independent of gp120 conformation. Accordingly, we observed no significant difference in mAb 2G12 binding to mature and immature virions (Fig. 3A, Fig. S6A). However, mAb 2G12 binding was increased by 35% on immature virions containing a truncated gp41 CT, consistent with a previous report that truncation of the CT enhances mAb 2G12 binding to Env expressed on the cell surface [15].

MAb B4e8 recognizes the V3 loop in gp120. We observed that mAb B4e8 binding to immature particles was increased by 15% relative to mature HIV-1 virions (Fig. 3B, Fig. S6B). On CT-truncated particles, the 15% increase in binding to immature particles was retained. However, neither of these differences was statistically significant, suggesting that exposure of the mAb B4e8 epitope is not markedly altered during HIV-1 maturation. The data from these two conformation-independent antibodies also corroborates the conclusion from the immunoblot analysis that the levels of gp120 are not significantly different on mature vs. immature virions.

### Immature HIV-1 virions are not impaired for CD4 binding

**Figure 4:**
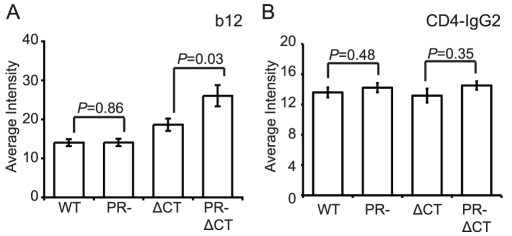
Binding of IgG1 b12 and CD4-IgG2 to HIV-1 particles. HIV-1 virions were processed and analyzed as described in the legend to Figure 2. N = 3 (b12 binding to ΔCT and PR-ΔCT), 4 (b12 binding to WT and PR-, CD4-IgG2); error bars represent SEM. (A) IgG1 b12 binding; (B) CD4-IgG2 binding.

A previous study reported that mature and immature HIV-1 particles are equally competent for binding to CD4+ T cells [13]. To test whether HIV-1 maturation alters the conformation of the CD4 binding site on gp120, we quantified the binding of mAb b12, which recognizes an epitope overlapping the CD4 binding site. MAb b12 bound mature and immature virions to an equivalent extent, suggesting that the region of gp120 recognized by this antibody is not structurally altered on the surface of immature HIV-1 particles (Fig. 4A, Fig. S7A). Curiously, the CT-deleted Env bound significantly more mAb b12 when present on immature vs. mature particles. Overall, these data suggest that the epitope recognized by mAb b12 is exposed to a similar extent on mature and immature HIV-1 particles and that the gp41 CT appears to modulate exposure of this epitope differentially on mature vs. immature particles.

To quantify CD4 binding to HIV-1 particles, we utilized CD4-IgG2, a fusion protein in which four copies of the V1 and V2 domains of human CD4 replace the heavy and light chain Fv portions of human IgG2. Because sCD4 has been shown to induce gp120 shedding to a different extent on mature vs. immature virions [20], we asked whether differential shedding contributed to the results obtained for CD4-IgG binding. For this purpose, we incubated HIV-1 particles with soluble CD4 (sCD4) and stained with mAb 2G12, whose binding should be unaffected by the conformational state of gp120. We found that while the mAb 2G12 signal decreased by 22% for mature HIV-1, the signal for immature HIV-1 decreased by only 11% (Fig. S8). Likewise, the mature CT-truncated virus signal decreased by 20%, and the immature tail-truncated virus signal decreased by only 3%. When the binding results were normalized by the observed levels of gp120 shedding, we observed no difference in binding of sCD4-IgG2 to mature and immature virions (Fig. 4B, Fig. S7B). Collectively, these results suggest that the CD4 binding site on gp120 is not structurally altered on the surface of immature virions. The data further demonstrate that the fusion impairment associated with immature viruses is not owing to a quantitative defect in CD4 binding.

### Immature HIV-1 virions are competent for CD4-induced gp120 conformational changes

**Figure 5:**
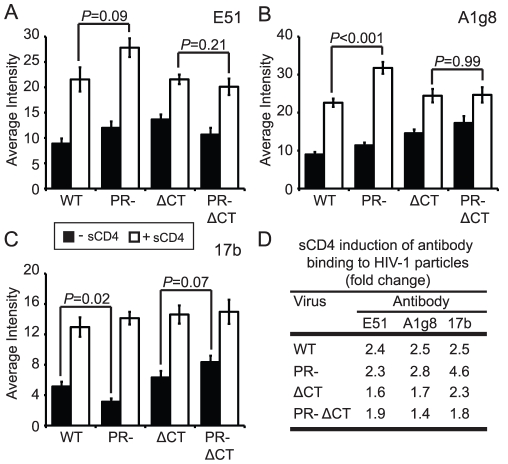
Analysis of sCD4-induced conformational changes on HIV-1 virions. HIV-1 virions were processed and analyzed as described in the legend to Figure 2. Filled bars: no sCD4. Open bars: with sCD4. N = 3 (ΔCT and PR-ΔCT), 4 (A1g8 and E51 binding to WT and PR-), 5 (17b binding to WT and PR-); error bars represent SEM. (A) mAb E51 binding; (B) mAb A1g8 binding; (C) mAb 17b binding; (D) quantitation of sCD4-induced antibody binding.

CD4 binding induces structural rearrangements in gp120, exposing epitopes recognized by the mAbs E51, A1g8, and 17b, which overlap the coreceptor-binding site in gp120 [21], [22]. We employed these antibodies in combination with soluble CD4 (sCD4), and taking into account the levels of sCD4-induced gp120 shedding, to quantify CD4-induced conformational changes on the surface of mature or immature HIV-1 particles. In the absence of sCD4, mAbs E51 and A1g8 binding to gp120 was approximately 20% greater on immature vs. mature HIV-1 particles (Fig. 5A and B, black bars, Fig. S9E and H). By contrast, binding of mAb 17b to immature particles was approximately 40% less than binding to mature particles (Fig. 5C, Fig. S9B). We also tested the effects of sCD4 on the binding of these antibodies (Fig. 5A–C, white bars, Fig. S9C,F,I). Binding of each antibody to immature virions was stimulated by sCD4 to a greater or equal extent vs. mature particles, with mAb 17b exhibiting the greatest increase (Fig. 5D). Truncation of the CT abolished the enhanced sCD4-induced binding of mAbs A1g8 and 17b to immature particles. Collectively, these results demonstrate that CD4 binding triggers exposure of some epitopes to an equal extent on immature and mature virions and other epitopes to a greater extent on immature virions. Furthermore, the involvement of the gp41 CT links the enhanced epitope exposure to the fusion impairment associated with immature HIV-1 particles.

### Immature HIV-1 particles exhibit enhanced exposure of specific epitopes in gp41

**Figure 6:**
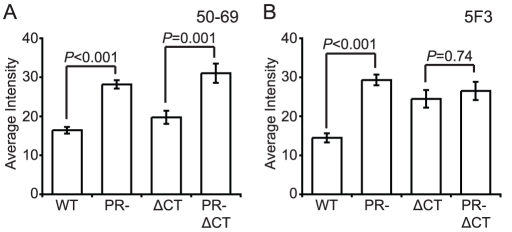
Antibody binding to non-neutralizing gp41 epitopes on HIV-1 virions. N = 3 (50–69 and 5F3 binding to ΔCT and PR-ΔCT), 4 (50–60 and 5F3 binding to WT and PR-); error bars represent SEM. (A) mAb 50–69 binding; (B) mAb 5F3 binding.

The fusion protein gp41 contains several epitopes that are exposed preferentially when Env is in a fusion-active conformation. MAb 50–69 binds at the C-terminal end of the N-terminal heptad repeat region of gp41 [23]. Binding of mAb 50–69 to immature particles was approximately 75% greater than to mature particles, a difference that was highly significant (Fig. 6A, Fig. S10A and B). Increased binding also was observed on particles lacking the gp41 CT. Thus, the Env protein on the surface of immature virions exhibits greater exposure of the mAb 50–69 epitope, but this conformational difference does not depend on the CT.

MAb 5F3 is a gp41-specific antibody that recognizes an epitope adjacent to the fusion peptide. We observed that mAb 5F3 binding to immature virions was approximately twice that of mature virions (Fig. 6B, Fig. S10C and D). When the CT was truncated, mAb 5F3 binding to mature particles was increased, but binding to mature and immature particles was equivalent. Thus, virion maturation masks the epitope recognized by mAb 5F3, and truncation of the gp41 CT abolishes this effect.

### The accessibility of the gp41 MPER is altered on immature HIV-1 particles

**Figure 7:**
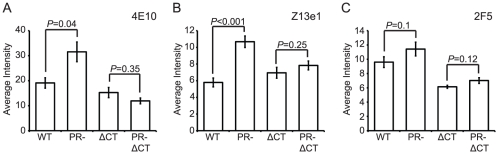
Analysis of neutralizing gp41 MPER epitopes. N = 3 (2F5 binding to ΔCT and PR-ΔCT, Z13e1), 4 (2F5 binding to WT and PR-, 4E10 binding to ΔCT and PR-ΔCT), 6 (4E10 binding to WT and PR-); error bars represent SEM. (A) mAb 4E10 binding; (B) mAb Z13e1 binding; (C) mAb 2F5 binding.

The MPER of gp41 is the target of several broadly neutralizing antibodies (bnAbs). The antigenic epitopes in this region of gp41 may be hidden from immune recognition, since the presence of such bnAbs is rare in infected patients. We tested the MPER-specific mAbs 2F5, Z13e1, and 4E10 for binding to mature and immature HIV-1 particles. Binding of mAbs 4E10 and Z13e1 to immature particles was greater than to mature virions (70% and 100% increase, respectively), and in both cases the increase was abolished by truncation of the gp41 CT (Fig. 7A and B, Fig. S1A–D). For mAb 2F5, slightly greater binding to immature particles was apparent, but the observed difference was not statistically significant (Fig. 7C, Fig. S1E and F).

On average, the MPER-specific mAbs 4E10 and Z13e1 bound immature particles approximately 1.5 to 2 times as well as mature particles when the median binding signals were compared. Examination of the distributions revealed that the differences resulted from increases in the percentage of immature particles at higher antibody binding levels (Fig. S1A and C). Analysis of mAb Z13e1-bound particles with an average intensity above 15 a.u. revealed a greater than 2-fold increase in binding to immature HIV-1 virions (Fig. S1D); analysis of mAb 4E10-bound particles with an average intensity above 50 a.u. revealed a 6-fold increase in binding to immature HIV-1 virions (Fig. S1B). We conclude that MPER-specific mAbs exhibit enhanced binding to immature HIV-1 particles, and the gp41 CT plays a role in controlling accessibility of MPER epitopes.

To test whether the increased binding was an effect of avidity caused by bivalent interaction with full-length IgG forms of the mAbs rather than increased affinity of the antibody combining site with the epitopes, we tested the binding to particles of Fab fragment forms of mAbs that were directly labeled with fluorophore. Consistent with the results using whole antibodies, Fab 4E10 exhibited significantly enhanced binding to immature vs. mature HIV-1 particles (Fig. S11), and the effect was abolished by truncation of the gp41 CT. By contrast, binding of Fab b12 did not exhibit a statistically significant difference. We conclude that MPER-specific antibodies exhibit preferential binding to immature HIV-1 particles and the gp41 CT contributes to the enhanced binding. These results reinforce the conclusion that the epitope recognized by mAb 4E10 is more accessible on immature HIV-1 particles.

## Discussion

In this study, we observed quantitative differences in epitope exposure on the surface of mature and immature HIV-1 particles using a novel single particle imaging-based binding assay. Several important epitopes exhibited increased exposure on the surface of immature HIV-1 particles. Specifically, exposure of the gp41 epitopes recognized by mAbs 50–69 and 5F3, and the MPER-specific mAbs Z13e1 and 4E10, were markedly increased on the surface of immature particles. By contrast, binding of mAb 17b, which recognizes a CD4-induced epitope, was lower for immature vs. mature particles. However, the accessibility of the CD4-induced epitope recognized by mAb 17b was generally increased on immature vs. mature particles. Taken together, the results indicate that the conformation of Env is altered on the surface of immature vs. mature HIV-1 particles, suggesting that the conformational of Env is altered during HIV-1 particle maturation.

Understanding the conformations of Env is of tremendous importance from an immunological perspective. HIV-1 rapidly evolves to evade the host antibody response, yet a current view is that an effective HIV-1 vaccine will require both humoral and cellular virus-specific responses. Despite considerable effort, attempts to elicit antibody responses that neutralize a wide range of HIV-1 isolates have been unsuccessful. Nonetheless, several human mAbs have been identified that exhibit broadly neutralizing activity, indicating that such antibodies can be produced in vivo, although they are rare. An area of intense study is the MPER region of gp41, which is the target of several bnAbs. In the current study, we show that the epitopes recognized by mAbs Z13e1 and 4E10 are bound more readily when present on immature particles, indicating that these epitopes become masked during HIV-1 particle maturation. Such conformational masking may represent an important mechanism of HIV-1 immune evasion, and immunization strategies targeting the MPER may benefit from focused approaches utilizing structurally engineered antigens informed by studies of immature HIV-1 particles. Therefore, a detailed understanding of the conformation of Env on immature particles could aid in the design of recombinant HIV-1 immunogens.

In the present study, we primarily analyzed the binding of full-length IgG antibodies rather than monovalent Fabs. Because IgG molecules are bivalent, their binding could be stabilized by avidity effects, which in turn could depend on the proximity of Env complexes on the virion surface. Our previous work showed that the Env proteins form a stable complex with the core within immature HIV-1 particles that depends on the gp41 CT. In mature virions, or particles lacking the gp41 CT, the Env trimers may be free to move laterally on the virion surface, thus allowing for patching of Env trimers. Thus, differences in antibody binding might be due, at least in part, to avidity effects resulting from altered trimer mobility in the viral membrane. To address this, we tested the binding of b12 and 4E10 Fab proteins, and the results agreed with the binding of the corresponding full-length IgG antibodies. Therefore, avidity effects seem unlikely to account for the enhanced antibody binding observed for immature particles, on which the patching of Env would be limited. Thus, our results are more consistent with the interpretation that immature particles exhibit increased exposure of specific epitopes vs. differential antibody-induced patching of Env trimers.

Previous work by our group and another demonstrated that immature HIV-1 particles are repressed for fusion with target cells by an activity that requires the gp41 CT [11], [13]. In the present study, we sought to test whether the repressed fusion of immature particles might be due to restricted conformational changes on the surface of immature particles. The tight association of both Env subunits with immature particles, observed even following detergent treatment, suggested that the Env might be locked in a “cloaked” fusion-inactive conformation owing to association of the gp41 CT with the highly stable Gag polyprotein lattice [8]. This cloaking mechanism would have the benefit of protecting the HIV-1 Env complex from neutralizing antibodies during the maturation process. Our results were surprising: exposure of several neutralization-sensitive Env epitopes was greater on immature particles. Several of the maturation-dependent conformational differences we observed were abolished by truncation of the gp41 CT, indicating that the CT couples Env conformation to particle maturation. CD4-induced binding of the gp120-specific antibody 17b also was enhanced on immature particles, and truncation of gp41 suppressed the enhanced binding. Therefore, our results establish that the gp41 CT alters the conformation of the Env ectodomains on immature particles, potentially interfering with the receptor-dependent conformational changes required for fusion. It is possible, however, that the enhanced sCD4-induced binding of 17b is unrelated to the fusion impairment associated with immature particles. Murakami *et al.* previously reported that immature HIV-1 particles are not impaired for binding of a mAb (NC-1) that recognizes the 6-helix bundle (6HB) conformation, which is thought to form at a late stage of the fusion process [13]. Specifically, the authors observed that addition of sCD4 induced equivalent binding of mAb NC-1 to mature and immature particles. While this result could suggest that the Env trimers on immature particles are competent for fusion-dependent conformational changes, it is not clear that mAb NC-1 binding to cell-free virions necessarily reflects a conformational change specific to fusion. Indeed, a recent study has shown that NC-1 recognizes other forms of gp41 besides the 6HB [24]. Therefore, it remains plausible that the differential binding of selected mAbs to mature and immature HIV-1 particles reflects conformational differences that contribute to the fusion impairment exhibited by immature HIV-1 virions.

Based on our results, we propose a model in which a strong Gag-Env association constrains the Env subunits, particularly gp41, into an exposed conformation, and that receptor engagement is insufficient to drive the additional conformational changes necessary for fusion. The model also implies that the MPER-specific bnAbs act by trapping gp41 in a conformational intermediate formed during particle maturation. Our results do not exclude other potential effects contributing to impaired fusion, such as altered physico-mechanical properties associated with immature particles bearing full-length Env proteins [14]. It should be noted that the present work employed a laboratory-adapted HIV-1 clone, which is likely hypersensitive to CD4. Fusion of viruses containing Env proteins from primary HIV-1 isolates is also regulated by maturation [25], and it will be important to extend the present studies to such Env proteins.

## Materials and Methods

### Cells and viruses

293T cells were cultured at 37°C and 5% CO_2_ in Dulbecco's Modified Eagle medium (DMEM; Cellgro) supplemented with fetal bovine serum (10%), penicillin (50 IU/mL), and streptomycin (50 µg/mL). The proviral DNA constructs used for the production of HIV-1 have been described previously [11] and are as follows: R9, wild-type HIV-1; R9.PR-, protease-defective HIV-1 containing a triple alanine substitution in the protease active site; R9Tr712, HIV-1 containing a truncation of the gp41 C-terminal 144 amino acids; R9Tr712.PR-, protease-defective HIV-1 containing a truncation of the gp41 C-terminal 144 amino acids. Viruses were produced by transient transfection of 293T cells in 10 cm dishes with 20 µg of proviral DNA and 7 µg of a GFP-Vpr fusion protein expression vector [26] using a calcium phosphate-based method [27]. Virus stocks were harvested 48 h after transfection and clarified through 0.45 µm syringe filters. Aliquots were buffered with 10 mM HEPES pH 7.3 prior to storage at −80°C. HIV-1 stocks were assayed for p24 by enzyme-linked immunosorbent assays (ELISAs) as previously described [8], [28], after boiling in SDS-PAGE loading buffer to solubilize the hyperstable immature particles.

### Antibodies and CD4 proteins

The following reagents were obtained through the NIH AIDS Research and Reference Reagent Program, Division of AIDS, NIAID, NIH: CD4-IgG2 from Progenics Pharmaceuticals; HIV-1 gp120 mAb 2G12 and HIV-1 gp41 mAbs 5F3, 2F5, and 4E10 from Dr. Hermann Katinger; HIV-1 gp120 mAbs F425 B4e8 and F425 A1g8 from Dr. Marshall Posner and Dr. Lisa Cavacini; HIV-1 gp120 mAb IgG1 b12 from Dr. Dennis Burton and Carlos Barbas; HIV-Ig from NABI and NHLBI; HIV-1 gp120 mAbs 17b and E51 from Dr. James E. Robinson; HIV-1 gp41 mAb 50–69 from Dr. Susan Zolla-Pazner; HIV-1 gp41 mAb IgG1 Z13e1 from Dr. Michael Zwick; sCD4-183 from Pharmacia, Inc.

### Production and labeling of purified recombinant Fabs

Amino acid sequences for Fabs b12 and 4E10 were obtained from the Protein Data Bank (PDB IDs 2NY7 and 2FX7). DNAs encoding the Fab protein sequences were designed and optimized for mammalian cell expression systems and synthesized commercially (GeneArt, Regensburg, Germany). The Fab DNAs were cloned into the pEE6.4 heavy chain expression vector (Lonza Group Ltd, Basel, Switzerland) with a recombinant stop codon placed before the constant region, to specify Fabs. The light chain cDNAs also were expressed in recombinant form using a mammalian cell optimized sequence that had been cloned into the pEE12.4 light chain vector (Lonza). The plasmids were transformed into DH5 strain *E. coli* cells for EndoFree Plasmid Maxi DNA preparation (Qiagen, Hilden, Germany). Transient transfection of each heavy and light chain combination for expression of Fab proteins in the serum-free HEK 293F cell expression system (Invitrogen, Carlsbad, CA) was accomplished using PolyFect reagent (Qiagen) according to the manufacturer's instructions. Supernatants were collected after 120 hours of expression and Fabs were purified using FPLC with a KappaSelect prepacked column (GE HealthCare Life Sciences, Piscataway, NJ) in D-PBS and concentrated in 15 mL centrifugal filter units with 30 kDa molecular weight cut-off (Millipore, Billerica, MA). A high level of purity of the Fabs was confirmed using a non-reducing SDS-PAGE and a Coomassie Blue stain (Invitrogen) that did not reveal contaminating proteins. The Fabs were shown to bind in ELISA as expected to HIV-1 virus-like particles or to recombinant gp120 molecules [29] to confirm their functionality before further study. Purified 4E10 and b12 Fabs were directly labeled using the Alexa Fluor 647 Microscale Protein Labeling Kit (Invitrogen) according to the manufacturer's instructions.

### Antibody binding assay for HIV-1 particles

HIV-1 virions were plated on poly-D-lysine coated dishes (MatTek). Virions were incubated with an Env-specific mAb (except in the case of polyclonal HIV-Ig) for two hours at room temperature, fixed in 2% paraformaldehyde for 15 min at room temperature, washed five times with PBS, incubated with a Cy5-conjugated anti-human IgG (Jackson ImmunoResearch Laboratories, Inc.) at a concentration of 14 µg/mL for one hour at room temperature, washed five times with PBS, mounted, and imaged using a Zeiss LSM 510 META inverted confocal microscope. Image acquisition was performed using a 63× objective lens with 2× optical zoom with line averaging for a 1024×1024 pixel image. GFP imaging was performed with an excitation wavelength of 488 nm and band pass 505–550 emission filter. Cy5 imaging was performed with an excitation wavelength of 633 nm and a long pass 650 emission filter. For preincubation with sCD4, viruses were incubated in suspension with 0.25 µg/mL 2-domain sCD4-183 for 30 min at room temperature prior to plating and addition of primary antibody. To optimize the assay for detecting differences in epitope exposure, each antibody was first titrated on wild-type and Env-deficient virions. Antibody concentrations giving minimal staining of Env-deficient virions and a non-saturated level of staining with wild-type virions were employed (Table S1). This allowed each antibody to be used within its dynamic range, so that any differences in binding to the viruses could be detected. Final antibody concentrations are provided in Table S1. CD4-IgG2 was used at 0.25 µg/mL. These concentrations were selected as the optimal concentrations that exhibited minimal background and were not saturating thus allowing detection of quantitative changes in epitope exposure.

Labeled 4E10 and b12 Fabs (1 µg/mL) were used in virus binding assays as described in the text. The only alteration to the protocol was eliminating the secondary antibody incubation and mounting immediately after the post-fixation washes.

### Image and data analysis

MetaMorph software (Molecular Devices) was used to quantify the average intensity of Cy5 staining for each GFP-positive particle. Particles were defined as adjacent groups of green pixels, or regions, above a background intensity of 20 graylevels in an area with a width and height between 0.2 and 0.8 microns. These regions were overlayed on the Cy5 image, and both the area and average signal intensity were calculated for each region. As can be seen in Figs. 1 and S2, not all Cy5 stained spots colocalize with a GFP region. We hypothesize that these spots are either HIV-1 particles that did not incorporate sufficient GFP-Vpr to be detected, or are microvesicles containing HIV-1 Env. Since Cy5 intensity was measured within regions defined by the presence of GFP, these GFP-negative particles were excluded from analysis. To verify that analyzing the average intensity for each particle would not skew the data, size distributions were generated for each virus type using the data from two independent mAb 4E10 and mAb b12 staining experiments (Fig. S12). These distributions, together with the absence of a correlation between the average GFP intensity per particle and particle area (Fig. S13B), showed that the apparent particle areas were not different for each type of HIV-1 virus used. In addition, there is no correlation between the average GFP intensity per particle and the average Cy5 (4E10) intensity per particle (Fig. S13A) and also no correlation between average Cy5 (4E10) intensity per particle and particle area (Fig. S13C). Of note, the observed higher levels of 4E10 binding occurred over the full range of particle sizes (Fig. S13C).

Data points are the median average intensity per particle of six independent fields (each contained approximately 200–600 particles) obtained from at least three independent experiments. These results were used in Wilcoxon rank-sum tests to evaluate statistical significance of observed differences. As a reference point, the particle size (Fig. S12) and the antibody binding histograms (Fig. S1,5,6,8,9,10) indicate the number of particles from one independent experiment (the combination of six images) used to generate the plots.

### Immunoblotting of viral lysates

Viruses particles were pelleted through a 20% sucrose cushion. Pellets were resuspended in SDS-PAGE sample buffer. Virion quantities were normalized based on p24 ELISA results prior to electrophoresis on 4%–20% SDS-PAGE gels (Bio-Rad) and transfer to nitrocellulose. Nitrocellulose was blocked in 5% nonfat dry milk in PBS containing 0.1% Tween-20. Primary antibodies were used as follows: gp120 mAb 2G12 (1 µg/mL), gp41 mAb 2F5 (1.25 µg/mL), CA 183-H12-5C (0.75 µg/mL). Secondary antibodies were donkey anti-human IgG IRDye800 (Rockland) and goat anti-mouse IgG DyLight680 (Thermo Scientific). Blots were scanned using the LI-COR Odyssey Imaging System, and bands were quantified using the instrument software.

### Histogram representation of antibody binding data

To generate example histograms for each antibody, the intensity values for each particle from six independent images acquired from one experiment were binned into 1 arbitrary unit (a.u.) bins. The percentage of particles falling within each bin was calculated based on the total number of particles, as indicated in the legend for each histogram, in the population. The y-axis scale was set to maximally visualize the WT, PR-, ΔCT, and PR- ΔCT virus populations. Therefore, the maximal value for the Env- population is indicated in parentheses in the top left corner of each plot.

### Cutoff analysis

To determine the percentage of particles stained above a cutoff intensity, as indicated by the white arrow and dotted line on the histograms, the average intensity values for each particle from six independent images acquired from one experiment (with the corresponding histogram representing one such experiment) were combined and analyzed as a single unit. The percentage of particles above the cutoff for each virus population in each experiment was calculated. These values were averaged for each virus and presented with the standard error of the mean. Each data point includes values from between three and six independent experiments.

### Assay of sCD4-induced gp120 shedding

To assess the level of gp120 shedding from mature and immature HIV-1, we quantified the level of mAb 2G12 binding following incubation with sCD4 under the conditions used for quantifying CD4-induced binding of mAb 17b. The HIV-1 viruses were plated on poly-D-lysine coated MatTek dishes, incubated with 0.25 µg/mL sCD4 for 30 min at room temperature, incubated with 1 µg/mL mAb 2G12 for 2 hours at room temperature, fixed in 2% paraformaldehyde for 15 min at room temperature, washed five times with PBS, incubated with a Cy5-conjugated anti-human IgG for one hour at room temperature, washed five times with PBS, mounted, and imaged. Image acquisition and data analysis were performed exactly as described in the text.

## Supporting Information

Figure S1 **Distribution of antibody binding intensities for mAbs 4E10, Z13e1, and 2F5.** (A) mAb 4E10. (C) mAb Z13e1. (E) mAb 2F5. The particles analyzed from six fields imaged during one independent experiment were combined and binned into 1 a.u. bins for distribution analysis. The numbers in parentheses at the top left corner of the plots indicate the percent particles per bin value at which the Env- samples peaked. The numbers in the parentheses next to the virus types in the legend represent the number of particles in each distribution. The arrows and dashed line indicate the cutoff level used in panels B, D, and F. The cutoff levels were selected by visual inspection of the distributions with the intent to determine whether the antibody binding differences are altered at high levels of binding. (B) mAb 4E10. (D) mAb Z13e1. (F) mAb 2F5. The percentages of particles with intensity greater than 50 a.u. for 4E10, 15 a.u. for Z13e1, and 15 a.u. for 2F5 were calculated. This value represents the area under the curve for each virus that falls above the cutoff intensity. N = 3 (2F5 binding to ΔCT and PR- ΔCT, Z13e1), 4 (2F5 binding to WT and PR-, 4E10 binding to ΔCT and PR- ΔCT), 6 (4E10 binding to WT and PR-); error bars represent SEM.
(PDF)

Figure S2 **Imaging of mAb 4E10 stained mature and immature HIV-1 virions.** The median average intensity per particle of the PR- image is 1.4-fold higher than that of the wild-type image.
(PDF)

Figure S3 **Staining and distribution analysis of Env-deficient immature virions.** PR-Env- virions were stained with (A and C) mAb b12 (1 µg/mL) or (B and D) mAb 4E10 (0.25 µg/mL). The staining distributions were plotted (A and B). The numbers within the parentheses are the percent particles per bin values at which the Env- and PR-Env distributions peaked. The median average intensity per particle for each virus is shown in C and D.
(PDF)

Figure S4 **Immunoblotting of viral lysates to compare Env levels.** (A) Immunoblots of pelleted viral lysates with detection of gp41 (1.25 µg/mL mAb 2F5), gp120 (1 µg/mL mAb 2G12), and CA (0.75 µg/mL 183-H12-5C); (B) Quantification of relative band intensities using LI-COR Odyssey Imaging System software.
(PDF)

Figure S5 **Distribution of antibody binding intensities for HIV-Ig.** The particles analyzed from six fields imaged during one independent experiment were combined and binned into 1 a.u. bins for distribution analysis. The number in parentheses at the top left corner of the plot indicates the percent particles per bin value at which the Env- sample peaked. The values in the parentheses next to the virus types in the legend represent the number of particles in the corresponding distribution.
(PDF)

Figure S6 **Distribution of antibody binding intensities for mAbs 2G12 and B4e8.** The particles analyzed from six fields imaged in an individual experiment were combined and binned into 1 a.u. bins for distribution analysis. The numbers in parentheses at the top left corner of the plots indicate the percent particles per bin value at which the Env- samples peaked. The values in the parentheses next to the virus types in the legend represent the number of particles in the corresponding distribution. (A) mAb 2G12. (B) mAb B4e8.
(PDF)

Figure S7 **Distribution of antibody binding intensities for mAb b12 and CD4-IgG2.** The particles analyzed from six fields imaged during one independent experiment were combined and binned into 1 a.u. bins for distribution analysis. (A) mAb b12; (B) CD4-IgG2.
(PDF)

Figure S8 **Analysis of sCD4-induced gp120 shedding.** HIV-1 particles were incubated with sCD4 (0.25 µg/mL) for 30 min at room temperature before staining with 2G12 (1 µg/mL). Samples were treated identically as the samples stained with the CD4i antibodies with the exception of the primary antibody used. The values represent the percentages of 2G12 staining on the sCD4 treated sample relative to the untreated sample.
(PDF)

Figure S9 **Distribution of antibody binding intensities for mAbs 17b, E51, and A1g8.** The particles analyzed from six fields imaged during one independent experiment were combined and binned into 1 a.u. bins for distribution analysis. (A) Overlay of panels B and C. (B) mAb 17b without sCD4. (C) mAb 17b with sCD4. (D) Overlay of panels E and F. (E) mAb E51 without sCD4. (F) mAb E51 with sCD4. (G) Overlay of panels H and I. (H) mAb A1g8 without sCD4. (I) mAb A1g8 with sCD4.
(PDF)

Figure S10 **Distribution of antibody binding intensities for mAbs 5F3 and 50–69.** (A) mAb 5F3. (C) mAb 50–69. The particles analyzed from six fields imaged during one independent experiment were combined and binned into 1 a.u. bins for distribution analysis. The arrows and dashed lines indicate the cutoff levels used in panels B and D. (B) mAb 5F3. (D) mAb 50–69. The percentage of particles stained with an intensity greater than 25 a.u. was calculated. This value represents the area under the curve for each virus that falls above the cutoff intensity. N = 3 (50–69 and 5F3 binding to ΔCT and PR- ΔCT), 4 (50–69 and 5F3 binding to WT and PR-); error bars represent SEM.
(PDF)

Figure S11 **Binding of Alexa Fluor 647-labeled Fab fragments to HIV-1 virions.** HIV-1 virions containing GFP-Vpr were incubated with Alexa Fluor 647-labeled Fab fragments (1 µg/mL) and analyzed for fluorescence by confocal microscopy and Metamorph. The data were compiled from three independent experiments where at least six independent fields were evaluated for the median average intensity per particle. N = 3; error bars represent SEM. (A) Fab b12. (B) Fab 4E10.
(PDF)

Figure S12 **Distribution analysis of particle areas.** Particles for each virus type were binned according to particle area for one independent experiment. Two examples are shown for mAb b12 (A) and mAb 4E10 (B). The right panels are an overlay of the left two panels. Numbers in parentheses represent the number of particles in each distribution.
(PDF)

Figure S13 **GFP intensity, Cy5 intensity, and particle area correlation analysis for a mAb 4E10 binding experiment.** (A) Scatter plot of average GFP-Vpr signal intensity versus average Cy5 signal intensity showing a lack of correlation. (B) Scatter plot of average GFP-Vpr signal intensity versus particle area showing a lack of correlation. (C) Scatter plot of average Cy5 signal intensity versus particle area showing both a lack of correlation and high levels of mAb 4E10 binding to PR- virions over the full range of particle sizes.
(PDF)

Table S1 **Primary antibody concentrations used in antibody binding assays.**
(PDF)
