# Supplementary figures and images for: Maturation-Induced Cloaking of Neutralization Epitopes on HIV-1 Particles
Source: PLoS Pathog. 2011 Sep 8;7(9):e1002234. doi: 10.1371/journal.ppat.1002234 (PMC3169560; doi:10.1371/journal.ppat.1002234)

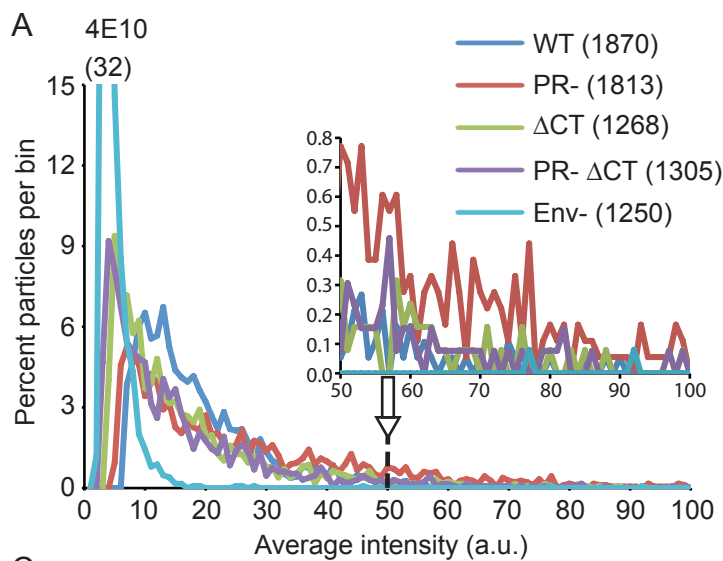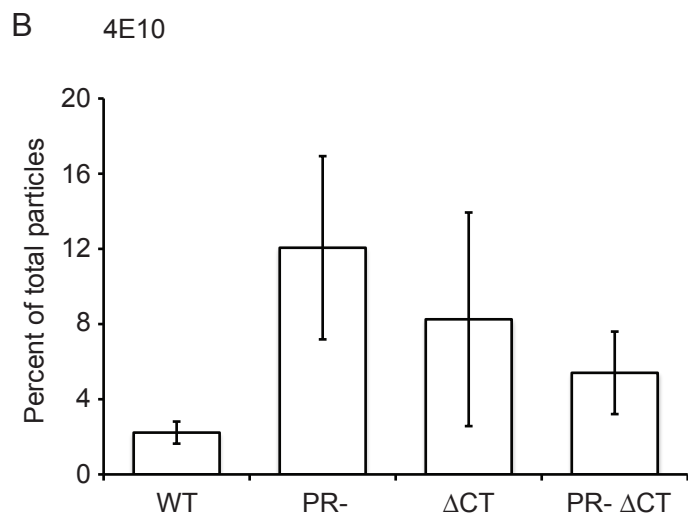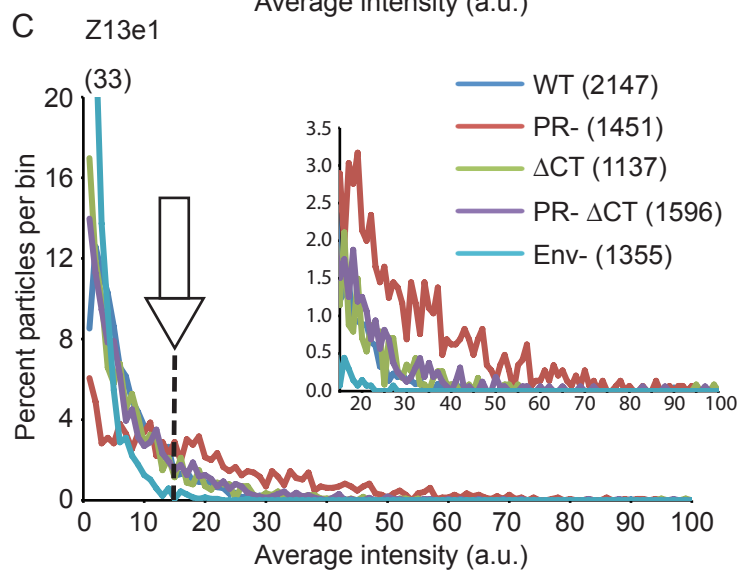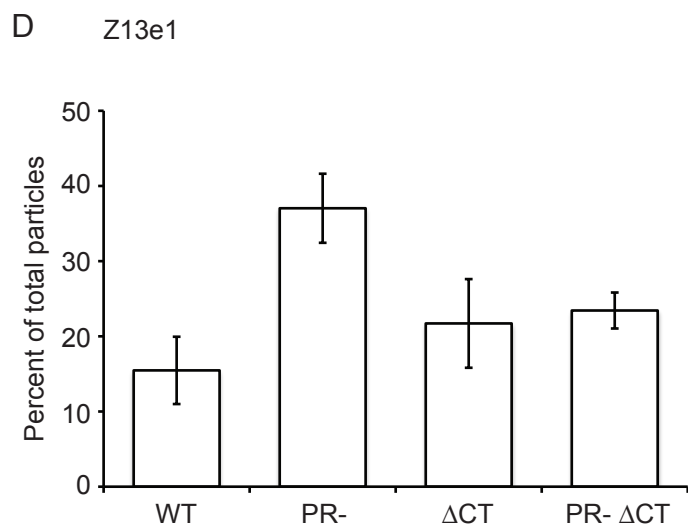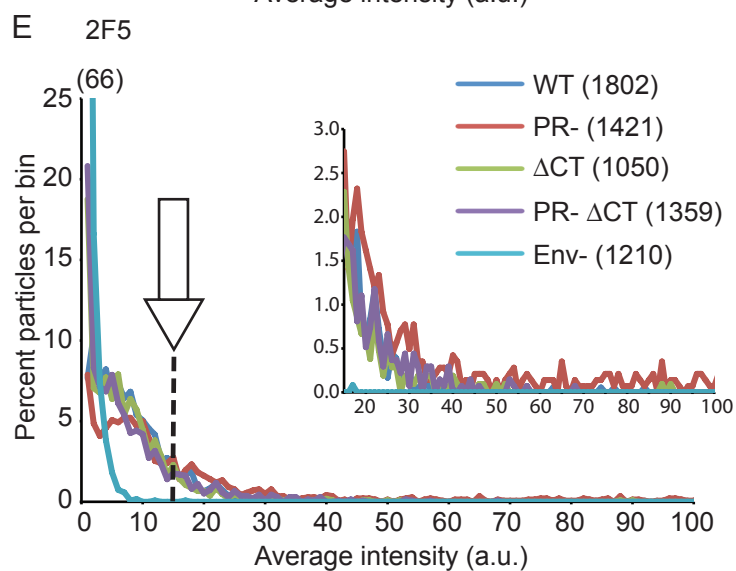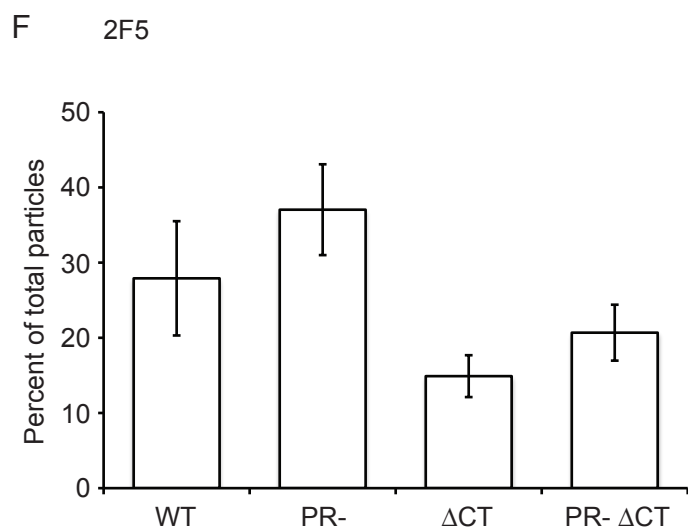

Supplement: Figure S1 — Distribution of antibody binding intensities for mAbs 4E10, Z13e1, and 2F5. (A) mAb 4E10. (C) mAb Z13e1. (E) mAb 2F5. The particles analyzed from six fields imaged during one independent experiment were combined and binned into 1 a.u. bins for distribution analysis. The numbers in parentheses at the top left corner of the plots indicate the percent particles per bin value at which the Env- samples peaked. The numbers in the parentheses next to the virus types in the legend represent the number of particles in each distribution. The arrows and dashed line indicate the cutoff level used in panels B, D, and F. The cutoff levels were selected by visual inspection of the distributions with the intent to determine whether the antibody binding differences are altered at high levels of binding. (B) mAb 4E10. (D) mAb Z13e1. (F) mAb 2F5. The percentages of particles with intensity greater than 50 a.u. for 4E10, 15 a.u. for Z13e1, and 15 a.u. for 2F5 were calculated. This value represents the area under the curve for each virus that falls above the cutoff intensity. N = 3 (2F5 binding to ΔCT and PR- ΔCT, Z13e1), 4 (2F5 binding to WT and PR-, 4E10 binding to ΔCT and PR- ΔCT), 6 (4E10 binding to WT and PR-); error bars represent SEM. (PDF) [file ppat.1002234.s001.pdf]

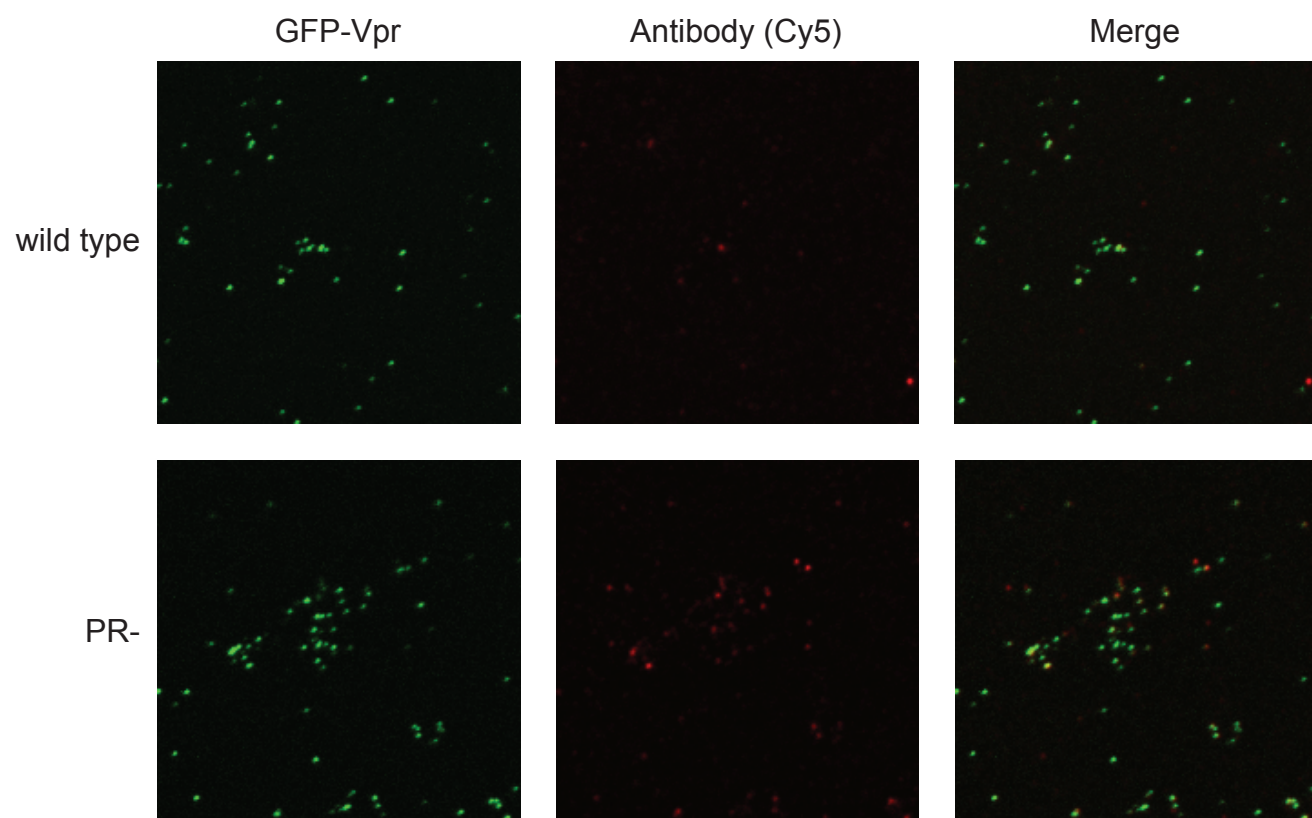

Supplement: Figure S2 — Imaging of mAb 4E10 stained mature and immature HIV-1 virions. The median average intensity per particle of the PR- image is 1.4-fold higher than that of the wild-type image. (PDF) [file ppat.1002234.s002.pdf]

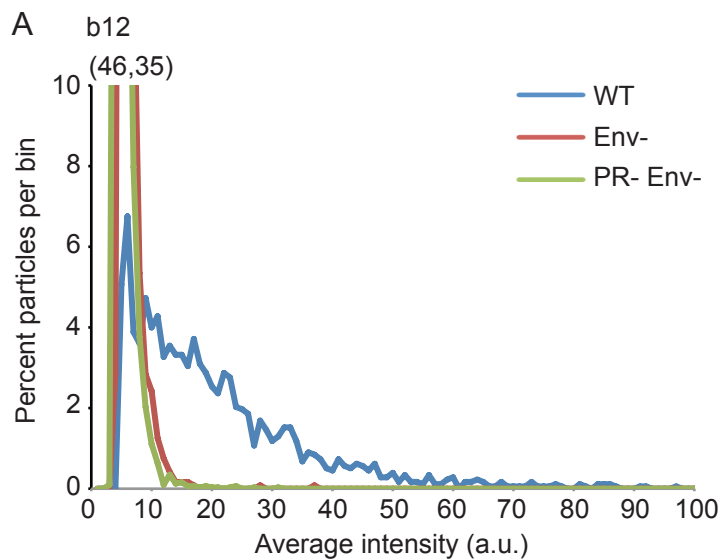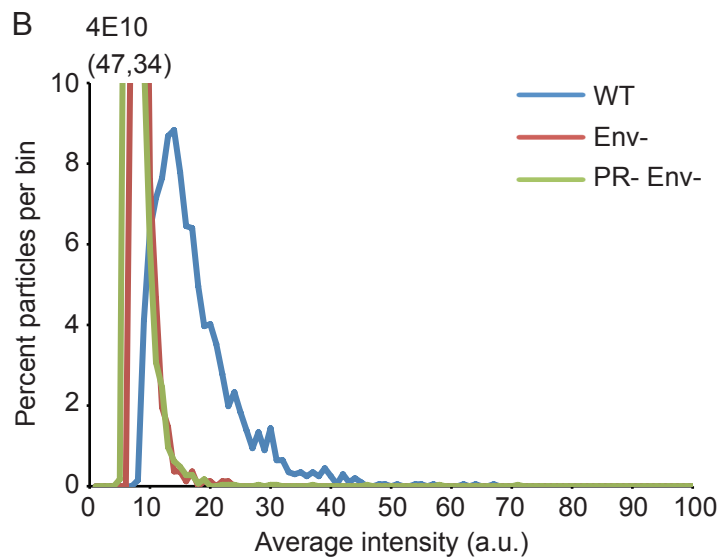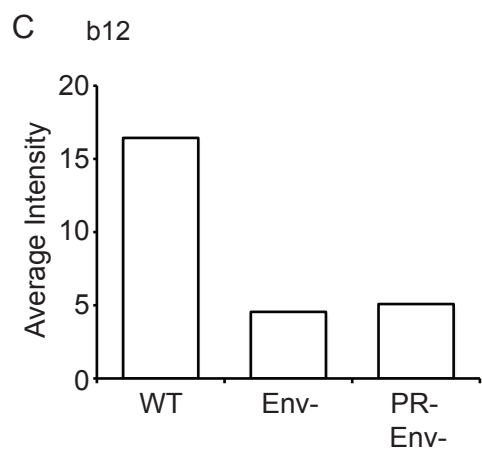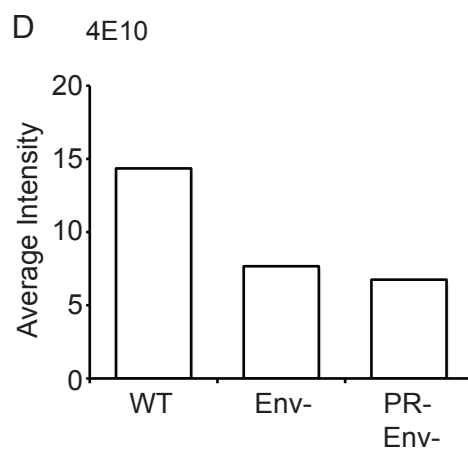

Supplement: Figure S3 — Staining and distribution analysis of Env-deficient immature virions. PR-Env- virions were stained with (A and C) mAb b12 (1 µg/mL) or (B and D) mAb 4E10 (0.25 µg/mL). The staining distributions were plotted (A and B). The numbers within the parentheses are the percent particles per bin values at which the Env- and PR-Env distributions peaked. The median average intensity per particle for each virus is shown in C and D. (PDF) [file ppat.1002234.s003.pdf]

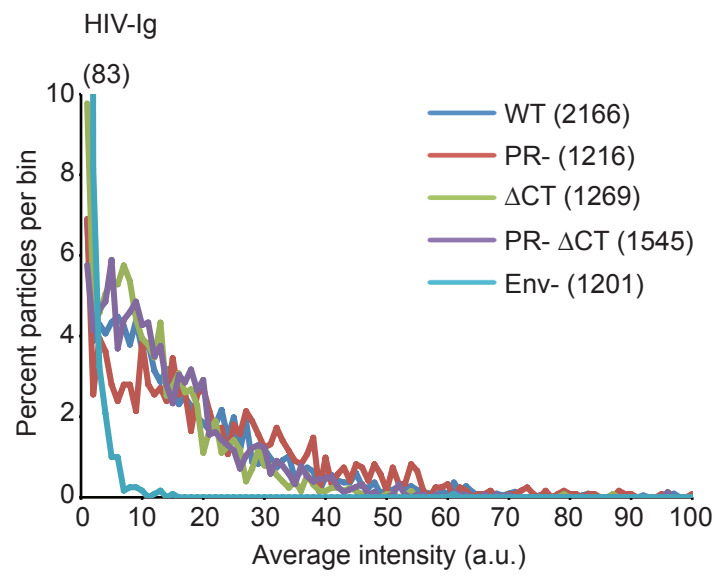

Supplement: Figure S5 — Distribution of antibody binding intensities for HIV-Ig. The particles analyzed from six fields imaged during one independent experiment were combined and binned into 1 a.u. bins for distribution analysis. The number in parentheses at the top left corner of the plot indicates the percent particles per bin value at which the Env- sample peaked. The values in the parentheses next to the virus types in the legend represent the number of particles in the corresponding distribution. (PDF) [file ppat.1002234.s005.pdf]

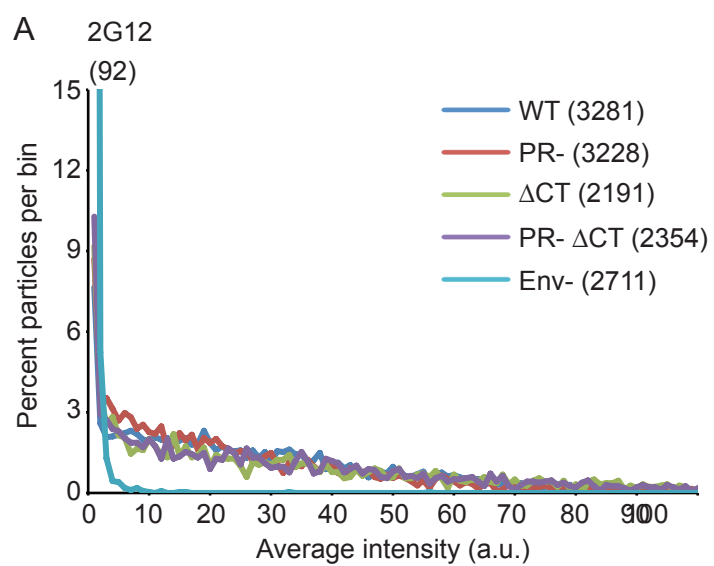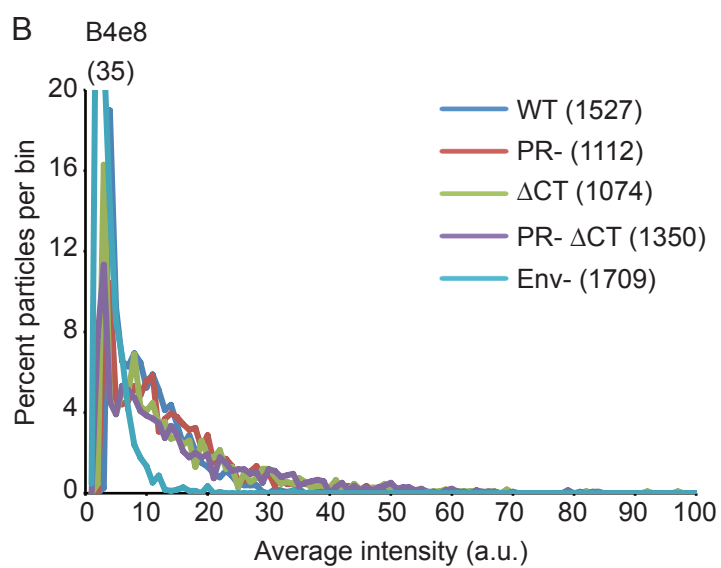

Supplement: Figure S6 — Distribution of antibody binding intensities for mAbs 2G12 and B4e8. The particles analyzed from six fields imaged in an individual experiment were combined and binned into 1 a.u. bins for distribution analysis. The numbers in parentheses at the top left corner of the plots indicate the percent particles per bin value at which the Env- samples peaked. The values in the parentheses next to the virus types in the legend represent the number of particles in the corresponding distribution. (A) mAb 2G12. (B) mAb B4e8. (PDF) [file ppat.1002234.s006.pdf]

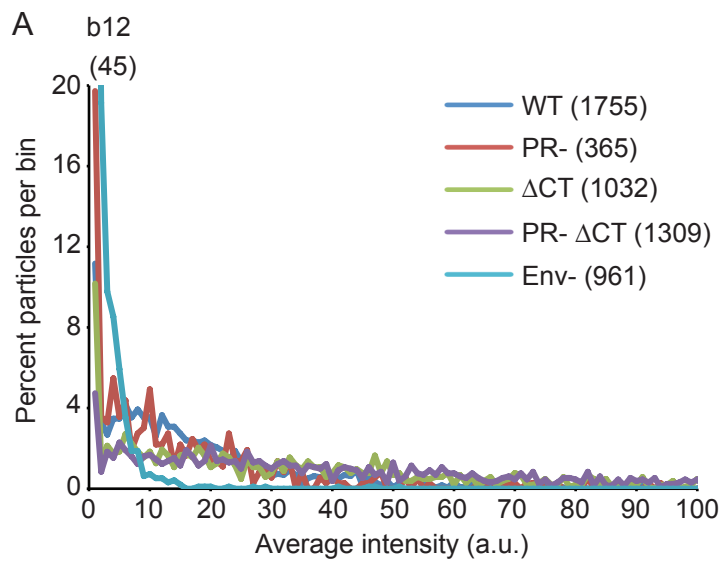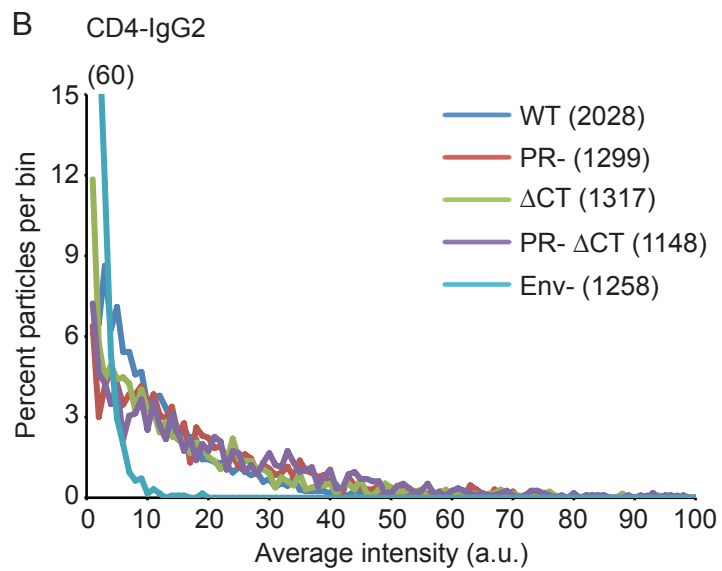

Supplement: Figure S7 — Distribution of antibody binding intensities for mAb b12 and CD4-IgG2. The particles analyzed from six fields imaged during one independent experiment were combined and binned into 1 a.u. bins for distribution analysis. (A) mAb b12; (B) CD4-IgG2. (PDF) [file ppat.1002234.s007.pdf]

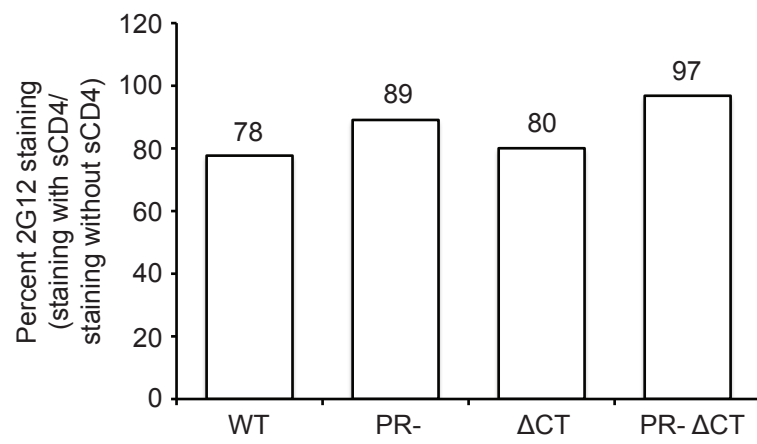

Supplement: Figure S8 — Analysis of sCD4-induced gp120 shedding. HIV-1 particles were incubated with sCD4 (0.25 µg/mL) for 30 min at room temperature before staining with 2G12 (1 µg/mL). Samples were treated identically as the samples stained with the CD4i antibodies with the exception of the primary antibody used. The values represent the percentages of 2G12 staining on the sCD4 treated sample relative to the untreated sample. (PDF) [file ppat.1002234.s008.pdf]

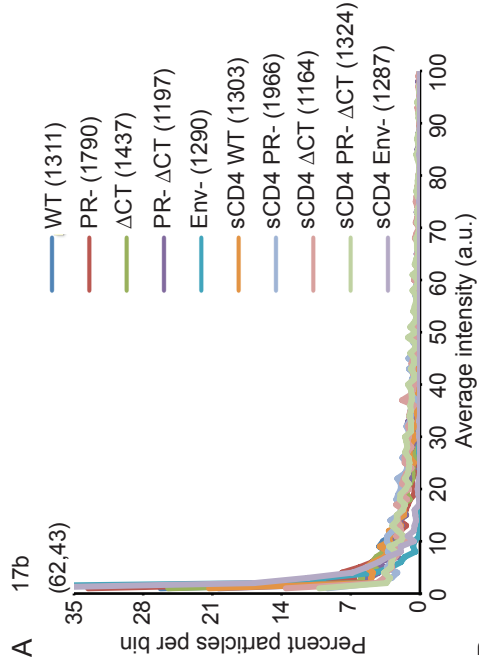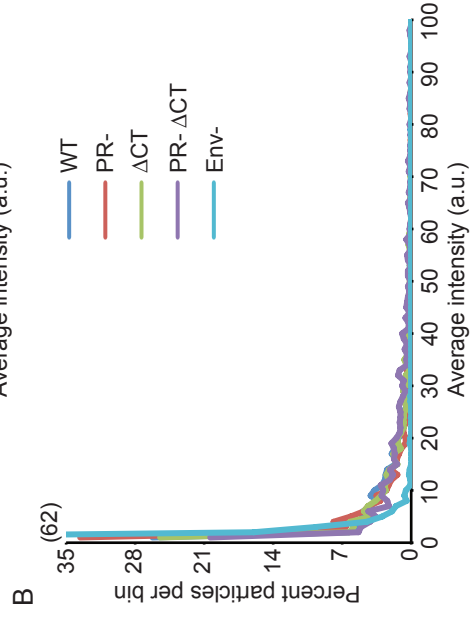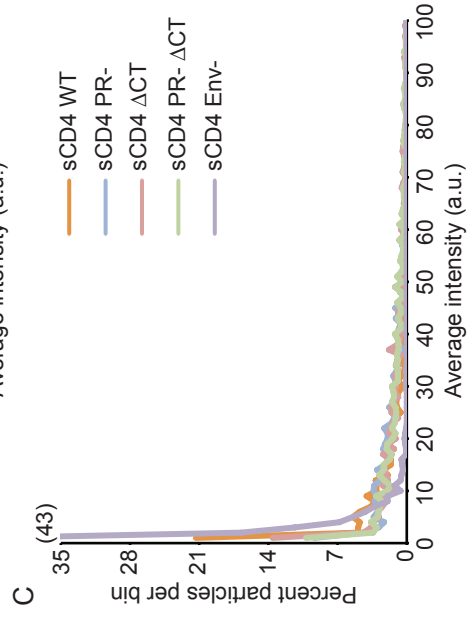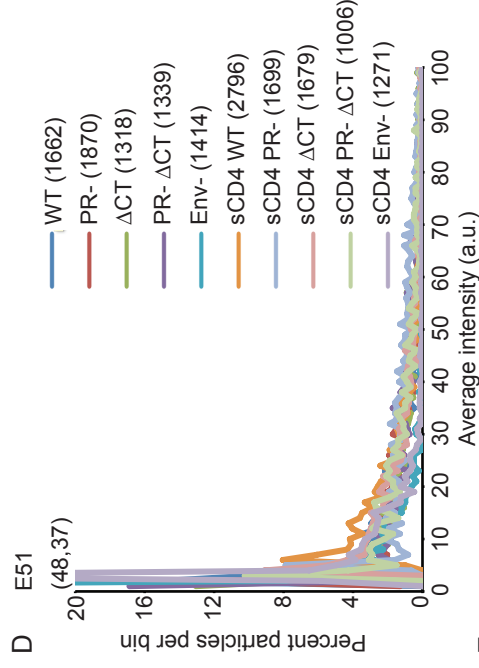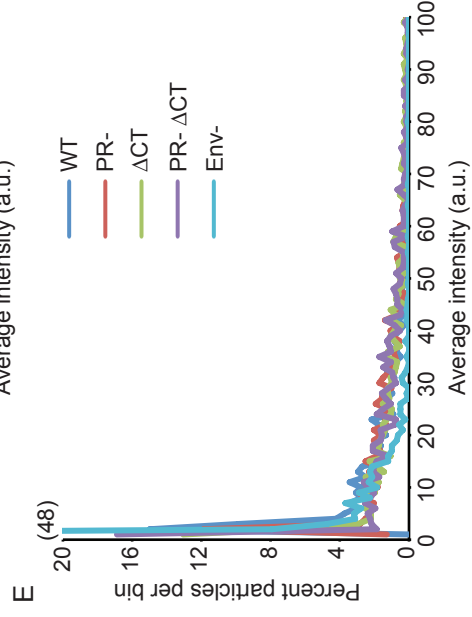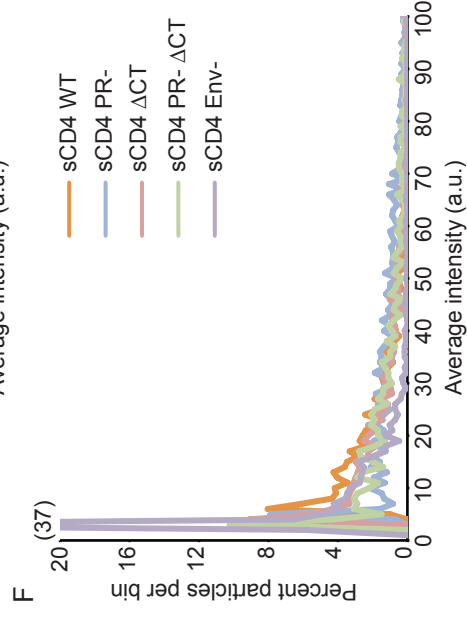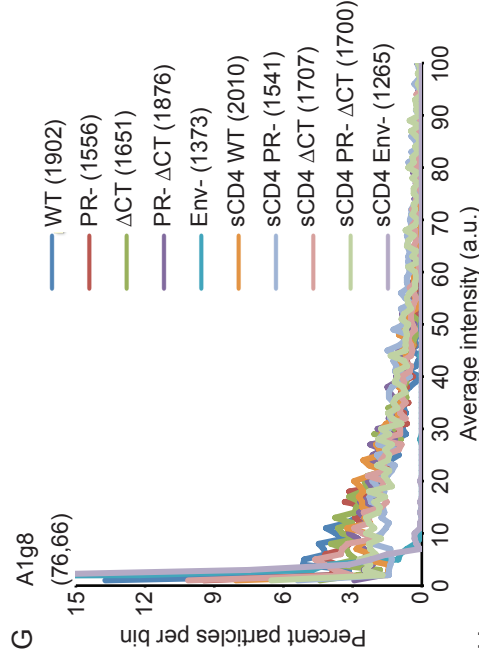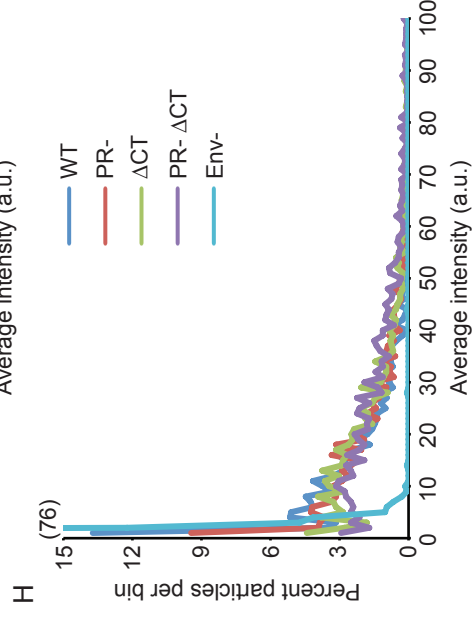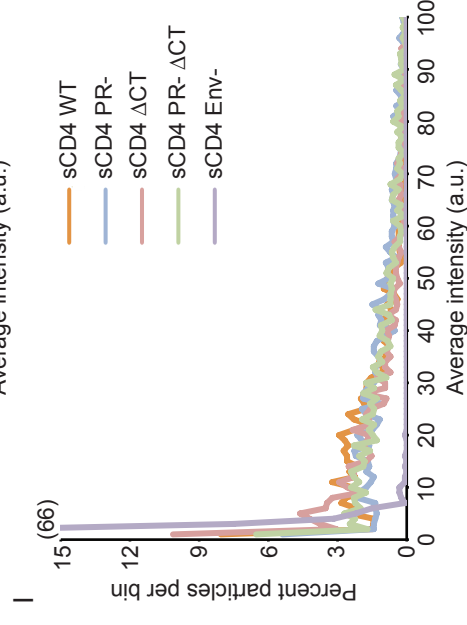

Supplement: Figure S9 — Distribution of antibody binding intensities for mAbs 17b, E51, and A1g8. The particles analyzed from six fields imaged during one independent experiment were combined and binned into 1 a.u. bins for distribution analysis. (A) Overlay of panels B and C. (B) mAb 17b without sCD4. (C) mAb 17b with sCD4. (D) Overlay of panels E and F. (E) mAb E51 without sCD4. (F) mAb E51 with sCD4. (G) Overlay of panels H and I. (H) mAb A1g8 without sCD4. (I) mAb A1g8 with sCD4. (PDF) [file ppat.1002234.s009.pdf]

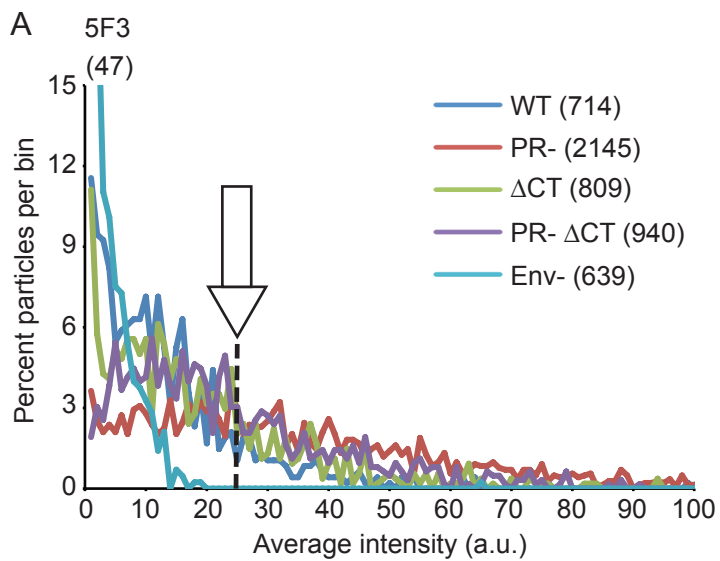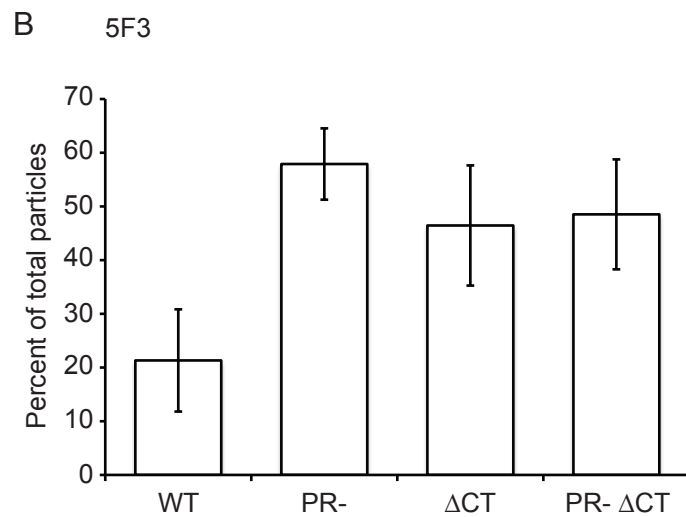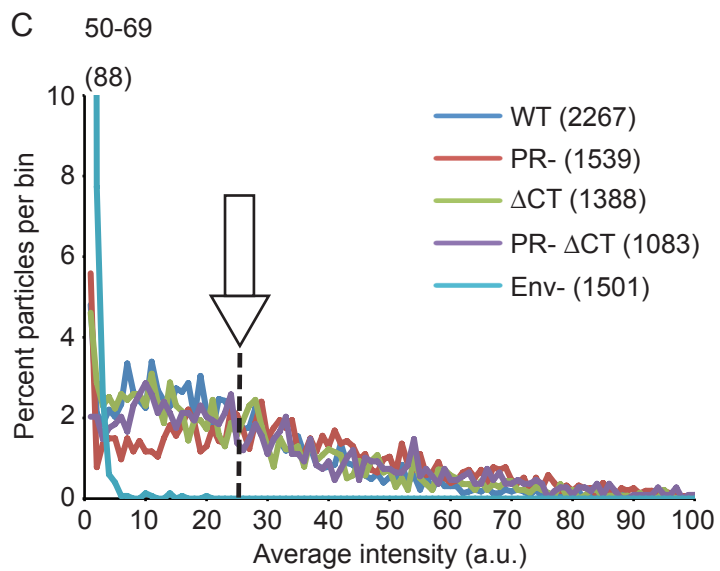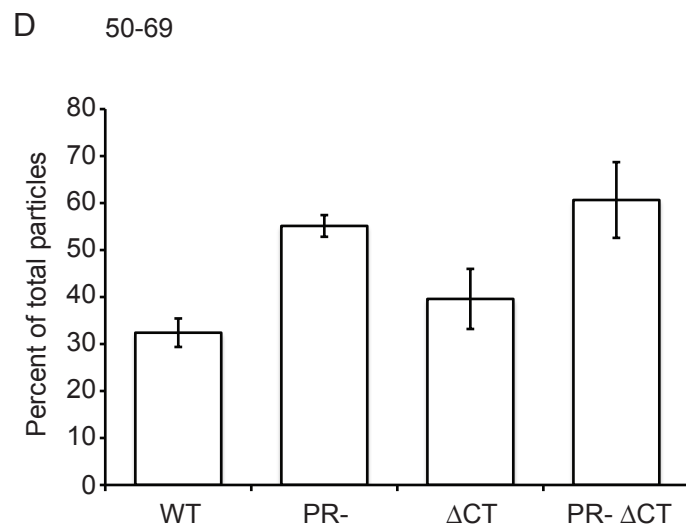

Supplement: Figure S10 — Distribution of antibody binding intensities for mAbs 5F3 and 50–69. (A) mAb 5F3. (C) mAb 50–69. The particles analyzed from six fields imaged during one independent experiment were combined and binned into 1 a.u. bins for distribution analysis. The arrows and dashed lines indicate the cutoff levels used in panels B and D. (B) mAb 5F3. (D) mAb 50–69. The percentage of particles stained with an intensity greater than 25 a.u. was calculated. This value represents the area under the curve for each virus that falls above the cutoff intensity. N = 3 (50–69 and 5F3 binding to ΔCT and PR- ΔCT), 4 (50–69 and 5F3 binding to WT and PR-); error bars represent SEM. (PDF) [file ppat.1002234.s010.pdf]

A Fab b12

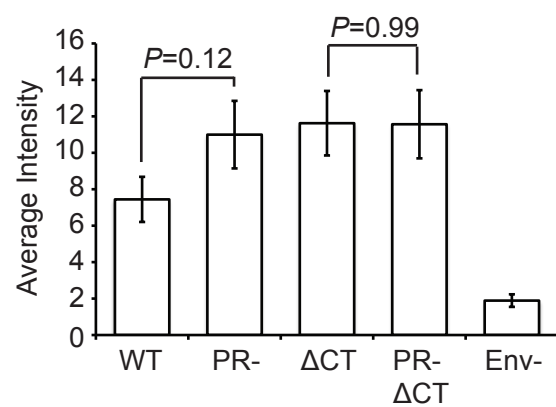

B Fab 4E10

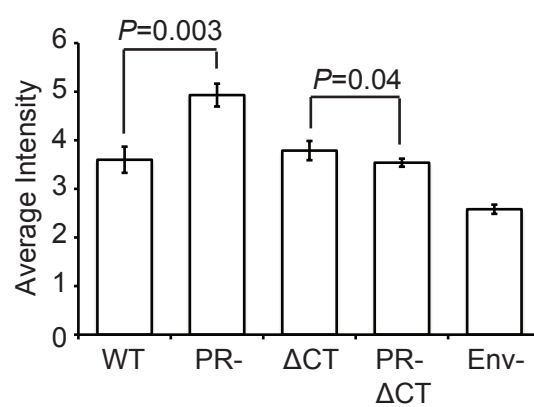

Supplement: Figure S11 — Binding of Alexa Fluor 647-labeled Fab fragments to HIV-1 virions. HIV-1 virions containing GFP-Vpr were incubated with Alexa Fluor 647-labeled Fab fragments (1 µg/mL) and analyzed for fluorescence by confocal microscopy and Metamorph. The data were compiled from three independent experiments where at least six independent fields were evaluated for the median average intensity per particle. N = 3; error bars represent SEM. (A) Fab b12. (B) Fab 4E10. (PDF) [file ppat.1002234.s011.pdf]

A b12

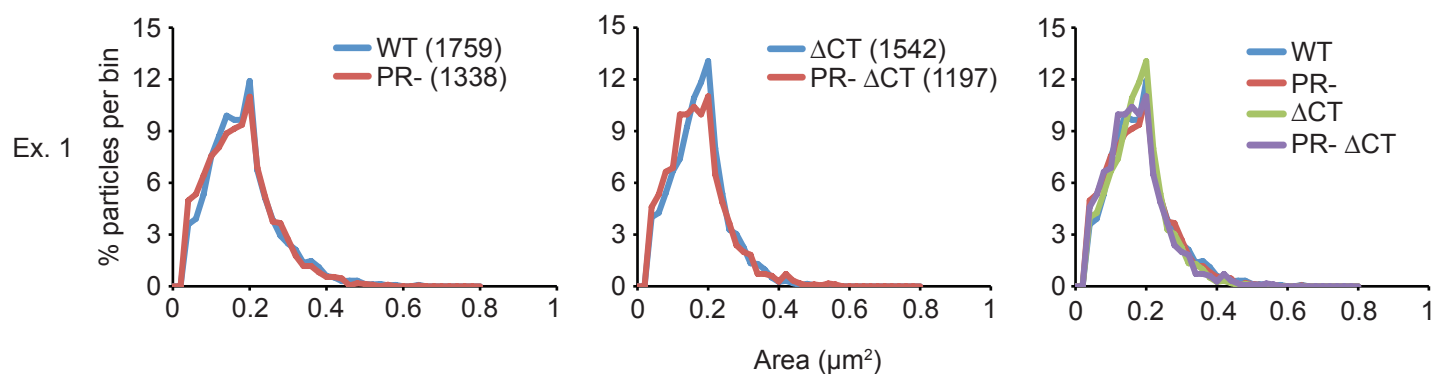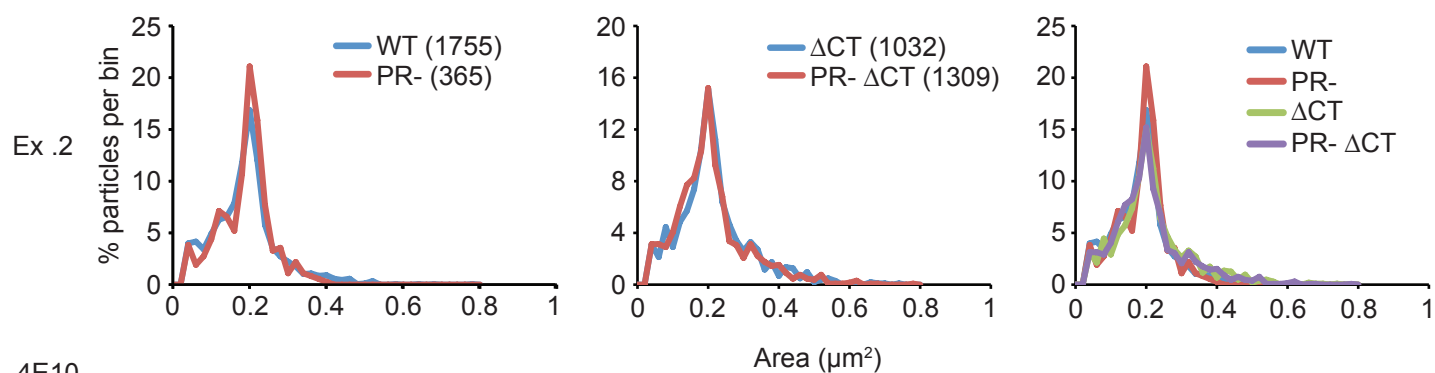

B 4E10

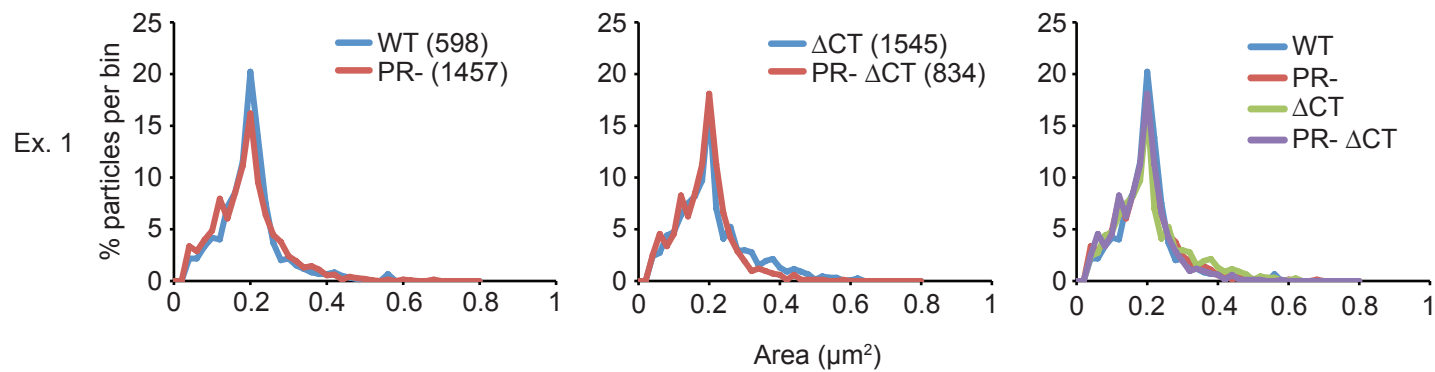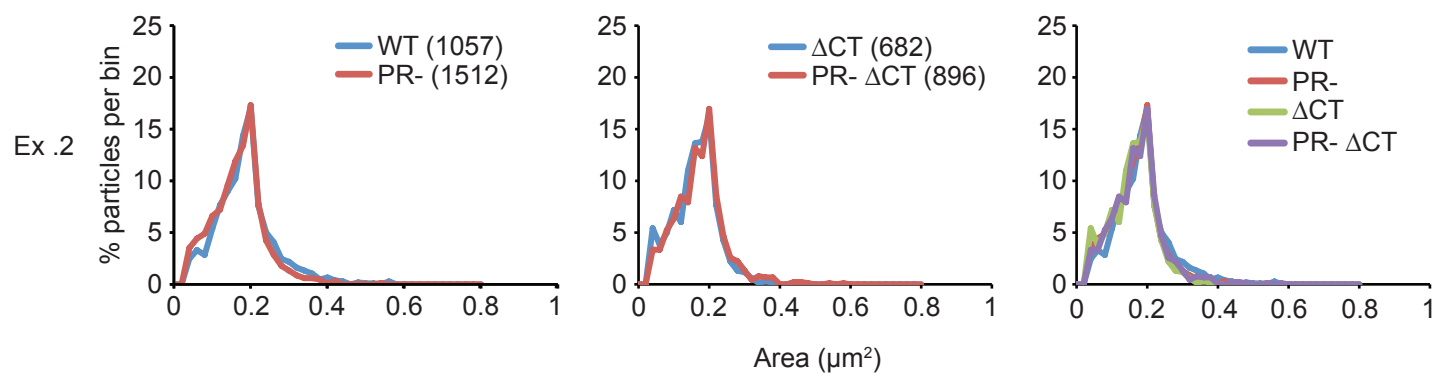

Supplement: Figure S12 — Distribution analysis of particle areas. Particles for each virus type were binned according to particle area for one independent experiment. Two examples are shown for mAb b12 (A) and mAb 4E10 (B). The right panels are an overlay of the left two panels. Numbers in parentheses represent the number of particles in each distribution. (PDF) [file ppat.1002234.s012.pdf]

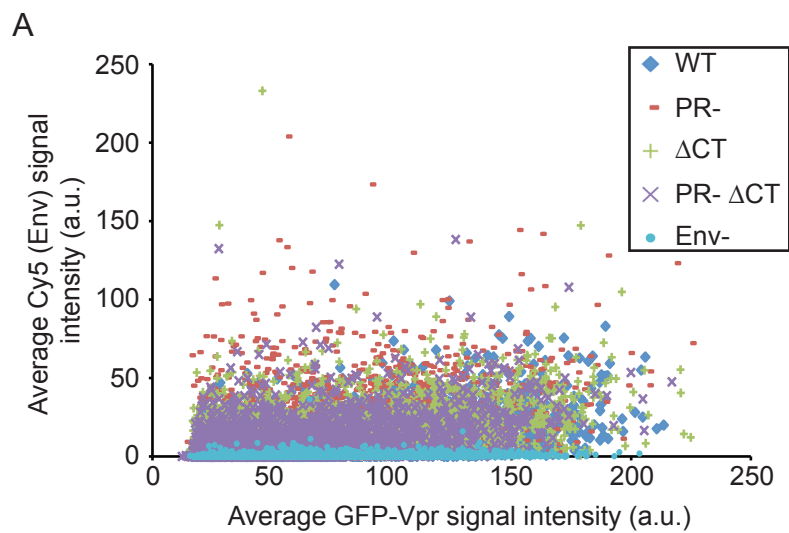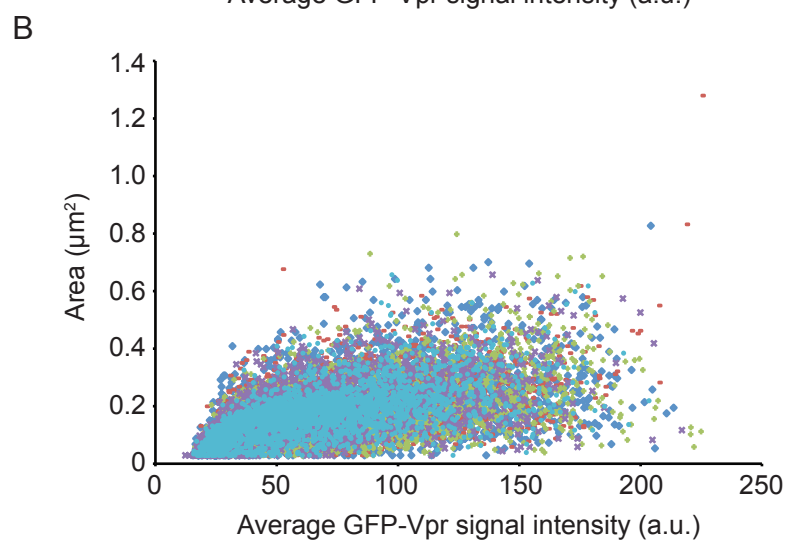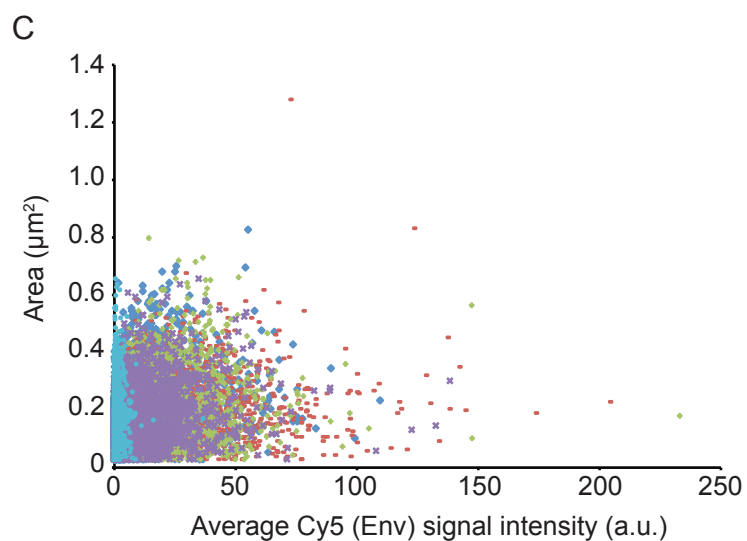

Supplement: Figure S13 — GFP intensity, Cy5 intensity, and particle area correlation analysis for a mAb 4E10 binding experiment. (A) Scatter plot of average GFP-Vpr signal intensity versus average Cy5 signal intensity showing a lack of correlation. (B) Scatter plot of average GFP-Vpr signal intensity versus particle area showing a lack of correlation. (C) Scatter plot of average Cy5 signal intensity versus particle area showing both a lack of correlation and high levels of mAb 4E10 binding to PR- virions over the full range of particle sizes. (PDF) [file ppat.1002234.s013.pdf]
