# Supplementary material for: Maturation-Induced Cloaking of Neutralization Epitopes on HIV-1 Particles
Source: PLoS Pathog. 2011 Sep 8;7(9):e1002234. doi: 10.1371/journal.ppat.1002234 (PMC3169560; doi:10.1371/journal.ppat.1002234)
Supplement: Figure S4 — Immunoblotting of viral lysates to compare Env levels. (A) Immunoblots of pelleted viral lysates with detection of gp41 (1.25 µg/mL mAb 2F5), gp120 (1 µg/mL mAb 2G12), and CA (0.75 µg/mL 183-H12-5C); (B) Quantification of relative band intensities using LI-COR Odyssey Imaging System software. (PDF) [file ppat.1002234.s004.pdf]

A

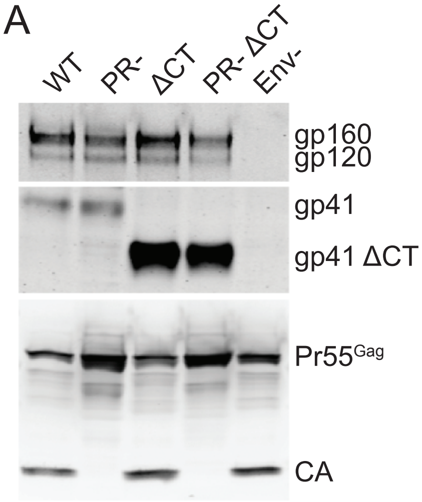

B

Quantification of band intensities relative to WT (fold change)

	gp41	gp120	gp160
WT	1	1	1
PR-	1.16	1	1
ΔCT	5.6	1.2	1
PR- ΔCT	5(0.9)	1(0.8)	0.8(0.8)

() indicates fold change relative to ΔCT
