# Supplementary material for: Maturation-Induced Cloaking of Neutralization Epitopes on HIV-1 Particles
Source: PLoS Pathog. 2011 Sep 8;7(9):e1002234. doi: 10.1371/journal.ppat.1002234 (PMC3169560; doi:10.1371/journal.ppat.1002234)
Supplement: Table S1 — Primary antibody concentrations used in antibody binding assays. (PDF) [file ppat.1002234.s014.pdf]

Table S1. Primary antibody concentrations used in antibody binding assays.

Antibody	Concentration (μg/mL)
17b	1.0
2F5	5.0
2G12	1.0
4E10	0.25
50-69	0.86
5F3	1.0
A1g8	1.0
CD4-IgG2	0.25
E51	1.0
F425 B4e8	5.0
HIV-Ig	5.0
IgG1 Z13e1	2.0
IgG2 b12	1.0
